# Supplementary material for: Beta band oscillations in motor cortex reflect neural population signals that delay movement onset
Source: eLife. 2017 May 3;6:e24573. doi: 10.7554/eLife.24573 (PMC5468088; doi:10.7554/eLife.24573)
Supplement: Figure 7—figure supplement 1—source data 1. — DOI: http://dx.doi.org/10.7554/eLife.24573.015 [file elife-24573-fig7-figsupp1-data1.docx]

**Figure 7—figure supplement 1—source data 1. Sample sizes and Kruskal Wallis test results for Figure 7—figure supplement 1.**

**Unit Properties of Chosen vs. Unchosen and Chosen+ vs. Chosen**

| **Monkey** | **Chosen** | **Unchosen** | **Classifier Wt** | **Beta Amplitude to-Spike Rate Slope** | **Mean Modulation (Hz)** | **Mean Firing Rate (Hz)** | **Beta Rhythmicity** |
| --- | --- | --- | --- | --- | --- | --- | --- |
| G | N = 104 | N = 251 | H = 32.42,  p = 1.24e-8 | H = 24.78, p = 6.413e-7 | H = .973, p = 0.324 | H = 14.40, p = 1.48e-4 | H = 0.229, p = 0.632 |
| C | N = 171 | N = 342 | H = 244.2,  p = 4.72e-55 | H = 145.0,  p=2.12e-33 | H = 1.195,  p = 0.274 | H = 139.6,  p=3.30e-32 | H = 0.364,  P = 0.546 |
| Comb. | N = 275 | N = 593 | H = 213.4, p=2.46e-48 | H = 132.3,  p=1.301e-30 | H = .004, p = 0.947 | H = 99.16,  p = 2.33e-23 | H = 0.016, p = 0.897 |
|  |  |  |  |  |  |  |  |
|  | **Chosen +** | **Chosen -** | **Classifier Wt** | **Beta Amplitude to-Spike Rate Slope** | **Mean Modulation (Hz)** | **Mean Firing Rate (Hz)** | **Beta Rhythmicity** |
| G | N = 85 | N = 19 | n/a | H = 35.72, p = 2.27e-9 | H = 0.0094, p = 0.923 | H = 7.13, p = 0.00756 | H = 1.56, p = 0.2116 |
| C | N = 170 | N = 1 | n/a | H = 2.50, p = 0.114 | H = 0.673, p = 0.412 | H = 2.965, p = 0.0851 | H = 0.059, p = 0.8079 |
| Comb. | N = 255 | N = 20 | n/a | H = 39.30, p = 3.64e-10 | H = 1.164, p = 0.281 | H = 3.702, p = 0.054 | H = 1.765, p = 0.184 |

Yellow highlighted indicates significant difference where Chosen > Unchosen or Chosen+ > Chosen -

Cyan highlighted indicates significant difference where Chosen < Unchosen or Chosen+ < Chosen –

All tests are Kruskal Wallis test for differences in median
