# Supplementary material for: Beta band oscillations in motor cortex reflect neural population signals that delay movement onset
Source: eLife. 2017 May 3;6:e24573. doi: 10.7554/eLife.24573 (PMC5468088; doi:10.7554/eLife.24573)
Supplement: Supplementary file 1. — DOI: http://dx.doi.org/10.7554/eLife.24573.016 [file elife-24573-supp1.docx]

**Supplementary Methods:**

**Chosen versus Unchosen Unit Analysis**

First, units were labeled as chosen or unchosen. Chosen units 1) successfully performed the PreMO and PostMO classification on their own (significantly lower scores for PreMO than PostMO in held-out CO and NF tasks with logistic regression trained on same training data used in Figure 7a, but with only 1 unit instead of the whole population) and 2) exhibited lower mean scores for ‘on-beta’ slow chunks than ‘off-beta’ slow chunks in both tasks. Unchosen units were all other units. Chosen units are also further broken down into Chosen+ and Chosen- units based on the sign of their weight (*β_1_*) in their individually trained logistic classifier.

Second, five unit metrics were computed:

1. **Weight** ($\beta_{1})$ from the individual unit logistic classifier used to predict PreMO vs. PostMO.

2. **Mean firing rates** of units during the 2.5-second epoch of the CO task

3. **Beta amplitude to firing rate slopes**, as in Figure 6c-h. Here, slopes are from the full set of CO data (not a subset)

4. **Mean task modulation during the CO task** – for the CO task, each units’ activity was averaged over each trial to give a mean trial trajectory over time. The maximum and minimum mean unit activity were subtracted, yielding the mean task modulation

5. **Beta rhythmicity** of each unit was computed by constructing two autocorrelation histograms per unit – one histogram was from time slices occurring during on-beta events and the other was from time slices occurring during off-beta events (as in (V.N. Murthy and Fetz, 1996). Next, an FFT was performed on each autocorrelation histogram. The difference in power between the on-beta and off-beta autocorrelation histogram at each frequency was computed. The power differences were then normalized to sum to 1 across frequencies to make units of different firing rates comparable, and the natural log was taken. Finally, the average of the logged power differences in the 20-45 Hz band were computed to yield the metric ‘Beta Rhythmicity’.
